# Reply to “Epidemiologic analysis of 8000 acute vertebral fractures: evolution of treatment and complications at 10-year follow-up”: food for thought outside the box

**DOI:** 10.1186/s13018-022-03280-5

**Published:** 2022-08-13

**Authors:** Rebecca Straessle, Corina Bello

**Affiliations:** 1 Emergency Department, Sonnenhof Hospital, Bern, Switzerland; 2 Department of Anesthesiology, Spitalregion Rheintal, Werdenberg, Sarganserland, Grabs, Switzerland; 3 Department of Anaesthesiology and Pain Medicine, Inselspital, Bern University Hospital, University of Bern, Bern, Switzerland

Dear Editor,

With great interest we read the retrospective cohort study by Bigdon et al. entitled “Epidemiologic analysis of 8000 acute vertebral fractures: evolution of treatment and complications at 10-year follow-up” [1]. The authors are to be congratulated on the extensive work they performed leading up to this large study. We would like to raise a number of points in connection with their work.

First, the authors state that fewer surgical therapies were performed before and after the 10-year study period. Nowadays, however, non-surgeons such as interventional neuroradiologists [2] also perform vertebroplasty and kyphoplasty [3]. Despite an increase in the need for early reoperation, overall and 5-year reoperation rates were comparable for non-surgical and surgical interventionists alike [3]. In a recent study, non-surgeons even experienced lower postoperative infection rates [3]. It might be interesting to compare the trend in surgical interventions for osteoporotic and acute vertebral fractures conducted by neuroradiologists in the same centre during the study period, in order to draw more accurate conclusions about changing treatment strategies and the changes in the number of interventions.

Second, implant failures, surgical site infections and postoperative neurological deterioration were reported by Bigdon et al. as the most common complications. However, current literature still mentions refractures in adjacent vertebrae as one of the major risks after vertebroplasty (at around 20%) [4]. It might be interesting to address this adverse effect and conduct further investigations on alternative determinants of surgical outcome as for example, the incidence of refractures in delayed surgical treatment after acute fractures, where literature is still scarce. Such findings should then be incorporated into personalised treatment decisions. Postoperative anti-osteoporotic treatment after vertebroplasty lowers the risk for refractures [5]. Also, preventive measures, as simple as optimised positioning, may help alleviate refracture incidence [6]. Moreover, the type of anaesthesia—such as local anaesthesia—might help prevent refractures [6]. Local anaesthesia for vertebroplasty or kyphoplasty is safe and regularly used in neurointerventional radiology [2]. Both patient positioning and anaesthesia type should be optimised by close interdisciplinary collaboration.

Next, the authors do not address preoperative optimisation for surgery, intraoperative care, and postoperative care as important influencers of patient outcome. They address persistent pain as a major complication. However, the time interval between vertebroplasty and follow-up impacts conclusions regarding pain improvement attributable to the surgical intervention. In osteoporotic thoracolumbar vertebral compression fractures treated with vertebroplasty, there is evidence for a significant reduction in pain scores in the acute setting (one day and two weeks post-intervention [7]) but not regarding pain three months after vertebroplasty [8] or chronic pain [9, 10]. Bigdon et al. only recognised persistent pain (defined as pain after 6 weeks [11]) as a major complication. It would be interesting to see such trends in pain scores in their cohort of patients including different fracture etiology. Also, strategies to proactively prevent chronic postoperative pain, such as preoperative analgesia, are vital [12] and deserve to be addressed. Improvements in quality of recovery from vertebroplasty could be achieved with pain relief based on a combination of medications with various mechanisms of action [12]. The use of local anaesthesia in the preoperative setting, as well as intraoperatively in combination with monitored anaesthesia care, has significant effects on orthopaedic outcome, overall pain scores, time to recovery and mobilisation, and is crucial in the multimorbid elderly generation [13]. Moreover, like local anaesthesia applied by anaesthetists, vertebral cancellous bone infiltration applied by surgeons may also alleviate intra- and postoperative pain [14]. Therefore, it is paramount to end silo thinking. We must create interdisciplinary enhanced-recovery-after-surgery protocols and standardise shared decision-making processes for preoperative and intraoperative patient-centred treatment.

## Data Availability

Not applicable.
